# Impact of Inertial Training on Muscle Strength and Quality of Life in Breast Cancer Survivors

**DOI:** 10.3390/ijerph19063278

**Published:** 2022-03-10

**Authors:** Alicja Naczk, Tomasz Huzarski, Janusz Doś, Magdalena Górska-Doś, Piotr Gramza, Ewa Gajewska, Mariusz Naczk

**Affiliations:** 1 Faculty of Physical Culture in Gorzow Wielkopolski, University School of Physical Education in Poznan, 66-400 Gorzow Wielkopolski, Poland; a.naczk@awf-gorzow.edu.pl (A.N.); j.dos@awf-gorzow.edu.pl (J.D.); m.gorska-dos@awf-gorzow.edu.pl (M.G.-D.); 2 Institute of Medical Sciences, Collegium Medicum, University of Zielona Gora, 65-417 Zielona Gora, Poland; t.huzarski@cm.uz.zgora.pl; 3 Department of Oncological Physiotherapy, Greater Poland Cancer Centre, 61-866 Poznan, Poland; 4 Association of Lubusz Innovation Network, 66-400 Gorzow Wielkopolski, Poland; p.gramza@slsi.pl; 5 Department of Developmental Neurology, Poznan University of Medical Sciences, 60-355 Poznan, Poland; ewagajewska@ump.edu.pl; 6 Institute of Health Sciences, Collegium Medicum, University of Zielona Gora, 65-417 Zielona Gora, Poland

**Keywords:** inertial training, mastectomy, breast cancer, quality of life

## Abstract

The aim of the study was to evaluate the impact of inertial training on the muscle strength, on breast-cancer-related lymphedema, and on quality of life in breast cancer survivors. After a mastectomy, 24 women (age, 66.2 ± 10.6 years) were randomized to a training (*n* = 12) or control group (*n* = 12). The training group performed inertial training twice per week for 6 weeks with a training load of about 70% of the maximal force. Before and after training, we tested the maximum force of shoulder flexors, extensors, abductors, and adductors; body composition; breast-cancer-related lymphedema; and disabilities of the arm, shoulder, and hand. Inertial training significantly improved the strength in all tested muscles (from 32 to 68%; effect size (ES) from 0.89 to 1.85 in the impaired limb and from 31 to 64%; ES from 0.86 to 1.57 in the unimpaired limb). However, changes in the control group were not significant. Quality of life improved following treatment; the disabilities of the arm, shoulder, and hand score decreased significantly by 24.5% (ES from—0.29 to 1.38), *p* ≤ 0.05 in the training group and by 3.99% (ES from −0.49 to 1.14) in the control group *p* > 0.05. Breast-cancer-related lymphedema and body composition did not change significantly after the intervention in either group. We recommend inertial training for increasing muscle strength and improving quality of life in breast cancer survivors.

## 1. Introduction

Breast cancer accounts for 24.5% of all cancers among women [1]. Although 80% of patients survive 5 years after treatment, their quality of life is significantly affected [2,3]. The most common problems observed after a mastectomy are deteriorated functional efficiency, reduced muscle mass and strength, lymphedema, pain around the treatment site, and limited range of motion in the shoulder joint [4]. Considering these consequences of a mastectomy procedure, an effective rehabilitation/training is usually needed. Strength training is one of the best workouts for people with low levels of functionality; strength training was shown to improve the quality of life, increase independence, and improve the health status [5,6]. Recent studies have indicated that strength training could be an effective form of rehabilitation that does not lead to lymphedema [7,8,9]. Moreover, Lane et al. [10] suggested that the intensity of routine exercise could potentially lead to improved lymph flow in the ipsilateral upper extremity, and perhaps, might prevent the development of breast-cancer-related lymphedema. Additionally, Wanhai and Armer [11] and Hasenoehrl et al. [12] concluded that supervised resistance exercise might be safe, feasible, and beneficial in patients with breast-cancer-related lymphedema and those at risk of developing it. Therefore, we hypothesized that properly supervised strength training might increase the muscle strength and improve the quality of life in women after a mastectomy.

One method of strength training involves inertial training, which is performed with a specialized device that imposes inertial resistance. This training differs from more traditional resistance modalities. During inertial training, great muscle tension remains both during concentric and eccentric contraction. In eccentric contraction, the muscle activity is greater than during concentric. Probably due to strong eccentric contraction during inertial exercises, great increases in muscle strength appear following training, and the effectiveness of inertial training can be greater than traditional resistance training [13]. The latest inertial devices allow electronic control and a display of training parameters, such as peak and mean force, power, work, time, and the number of repetitions [14]. Therefore, the device allows one to set precise loads and control muscle force during exercise. This control is particularly important for participants with poor health status that have low muscular efficacy.

To date, we lack data on the effectiveness and safety of inertial—eccentric exercises in breast cancer survivors after a mastectomy. However, inertial training has been used increasingly to improve the quality of life of individuals with limited functionality [15,16]. Studies on the efficacy of inertial training in young healthy subjects have indicated that it is a highly effective and safe training method [17,18]. Moreover, Maroto-Izquierdo et al. [13] concluded that inertial training triggered significantly greater skeletal muscle adaptations (i.e., strength, power, and muscle mass) compared to traditional, gravity-dependent resistance exercise paradigms. Taking into consideration existing knowledge about inertial training, we hypothesized that inertial training with low loads and high intensity might be an effective training method for women that underwent a mastectomy due to breast cancer. We hypothesized that this type of inertial training might increase the muscular strength and improve the quality of life in breast cancer survivors.

The present study aimed to evaluate the impact of inertial training on the strength of shoulder flexors, extensors, abductors, and adductors in breast cancer survivors after a mastectomy. In addition, we investigated whether training influenced the quality of life of participants and breast-cancer-related lymphedema.

## 2. Materials and Methods

### 2.1. Participants

**Figure 1:**
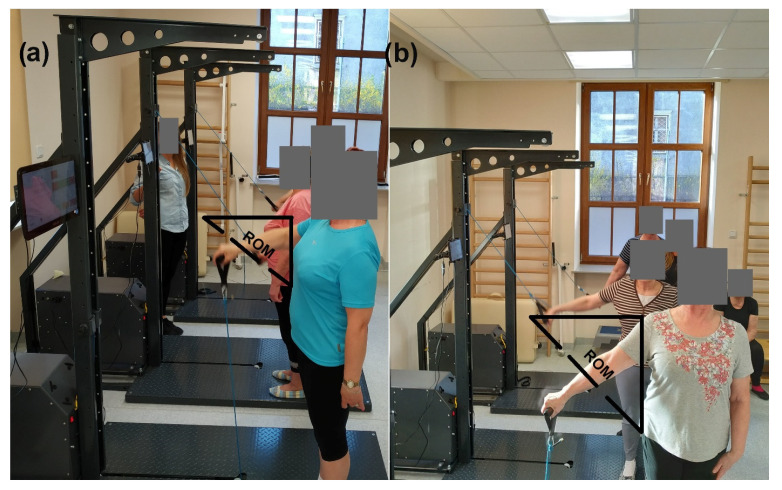
Subject positions during strength testing and training. The subjects’ positions (**a**) during flexion (first person) and during extension (second person); (**b**) during abduction (first person) and during adduction (second person). ROM—range of motion during flexion and extension (**a**) and during abduction and adduction (**b**).

All participants provided written informed consent to take part in the study. Moreover, the participants shown in Figure 1 provided written informed consent to allow their images to be published. All procedures were approved by the local ethics committee, with approval based on the Declaration of Helsinki, and all methods were carried out in accordance with the relevant guidelines and regulations.

A group of 30 women who underwent a mastectomy procedure attended an initial recruitment meeting, and all 30 women agreed to participate in the study. The participants were members of an association (Amazons Association, Gorzow Wielkopolski, Poland) of individuals that had experienced a mastectomy (average time from surgery: 12.1 years). Twenty-four women met the inclusion criteria: a partial or simple mastectomy was performed, and subjects received permission from their physician to participate in the experiment. Exclusion criteria: fractures in the prior 3 months, tendon or ligament injury in the prior 2 months, serious heart disease, cerebral palsy, and limb amputations. Therefore, the study finally included 24 women that had experienced a mastectomy (mean ± standard deviation: age, 66.2 ± 10.6 years, range 43–82 years; body mass, 72.7 ± 10.6 kg; height, 159 ± 5.2 cm). Among these, 17 had undergone a simple mastectomy and 7 had undergone partial mastectomy; an average of 15 lymph nodes were removed during surgery; more details are shown in Table 1.

During the research, none of the participants received radiotherapy or chemotherapy. Four women were using hormone therapy during the study.

The participants were randomly allocated into two groups: a training group (*n* = 12) and a control group (*n* = 12) with the chit method—a simple method of generating random sequence. For random allocation of 24 cases into two groups equally, 24 chits were prepared: writing “C” (for control group) on 12 chits and “T” (for training group) on 12 chits. After folding the chits, putting in a box and mixing well, every participant drew a single chit. The results of the draw were noted by researcher and participants were allocated into the training or control group.

The training group participated in 6 weeks of inertial training. The control group maintained normal daily activity.

Before and after training, all participants were evaluated to determine the strength of shoulder muscles, body composition, breast-cancer-related lymphedema, and disabilities of the arm, shoulder, and hand (DASH).

### 2.2. Strength of Shoulder Muscles Measurement—Primary Outcome

We used a Cyklotren inertial device (Inerion, Stanowice, Poland) to test the strength of the shoulder flexors, extensors, abductors, and adductors [14]. The Cyklotren measurements exhibit very high reproducibility (intraclass correlation coefficient [ICC] consistency ≥0.969, ICC agreement ≥ 0.965) [14]. Patients were tested in the standing position. Briefly, after a warm-up, each participant performed a 10 s maximal strength test consisting of shoulder flexion, extension, abduction, and adduction with the right and left arms separately, with a 2 min break between measurements. The positions of participants during strength testing and inertial training are shown in Figure 1. For all measurements, a 5 kg load was applied. The mean values for maximal force (N) from the impaired and unimpaired arms were used for further analysis.

It should be noted that the load applied during inertial training did not necessarily induce the working muscles to develop the same force. During inertial exercise, the force developed by muscles strongly depends on the movement velocity; in other words, greater movement velocities result in greater muscle loads, and consequently, greater force is developed (the force value is displayed electronically on the Cyklotren screen). The range of motion was approximately 90 degrees (where 0 degrees corresponded to the lowered arm, extended along the trunk). Data collection was preceded by a familiarization session.

### 2.3. Body Composition Measurements

We performed bioelectric impedance to evaluate the body composition of participants (Tanita 980 MC, Tokyo, Japan). The participants were asked to maintain a normal state of hydration, and they were not allowed to exercise, eat, or drink alcohol or caffeine for 12 h preceding the measurements. Measurements were conducted in the morning, according to the manufacturer’s guidelines. Fat mass, muscle mass, and water content measurements were used in further analyses.

### 2.4. Breast-Cancer-Related Lymphedema Evaluation

We performed bioimpedance spectroscopy to evaluate the influence of inertial training on breast-cancer-related lymphedema. This technique allows one to detect small changes in extracellular fluid and subclinical breast-cancer-related lymphedema. Thus, it provides subclinical detection of breast-cancer-related lymphedema, when swelling is not apparent. Measurements were performed with using L-Dex U400 unit (ImpediMed Limited, Pinkenba, Australia). The feasibility and clinical utility of implementing L-Dex measurements in routine breast cancer care were confirmed in previous studies [19,20]. For these measurements, patients were lying supine on a non-metallic surface. We used a standardized technique, described by Laidley and Anglin [19]. Measurements were taken with patients lying supine on a rehabilitation table. Electrodes were placed on the skin on the midline dorsal surface of the wrist at the level of the ulnar styloid process and on the skin on the midline anterior surface of the ankle at the level of the medial and lateral malleolus bones.

### 2.5. Disabilities of the Arm, Shoulder, and Hand Test (DASH)

Participants completed a DASH questionnaire to evaluate their ability to perform specific daily activities and quality of life. The DASH questionnaire is a standardized measure, which captures the patients’ own perspective of their upper-extremity health status [21]. The main part of the DASH is a thirty-item disability/symptom scale concerning the patient’s health status during the preceding week. The items ask about the degree of difficulty in performing different physical activities because of the arm, shoulder, or hand problem (21 items), the severity of each of the symptoms of pain, activity-related pain, tingling, weakness and stiffness (5 items), as well as the problem’s impact on social activities, work, sleep, and self-image (4 items). Each item has five response options (from 1—no problem to 5—biggest problem). The scores for all items are then used to calculate a scale score ranging from 0 (no disability) to 100 (most severe disability). The questionnaire was designed to allow participants to select the answers freely.

## 3. Workout

Inertial training was performed twice/week (Mondays and Thursdays, between 5:00 and 8:00 p.m.) for 6 weeks on the Cyklotren device (Inerion, Stanowice, Poland). Exercises were supervised by the same two researchers. Each training session included a warm-up and four sets of shoulder flexion, extension, abduction, and adduction exercises, performed with the right and left arms separately (16 sets for each arm). Each set lasted 15 s; a 2 min break was allowed between consecutive sets (without a rest period between the right and left arm exercises). A 5 kg load was applied to all training muscles. During training, participants developed 70% of the maximal force measured during the strength measurement (before training). During each set, the participants performed 12–14 reps (depending on the maximal strength). As the strength of participants increased following training, the number of repetitions increased. After 2 and 4 weeks of training, the maximal force was measured, and following muscle strength changes, the training loads were changed; participants developed 70% of the maximal force. The force developed during training was displayed electronically on the screen of the Cyklotren device. Cyklotren software allowed participants to set the upper and lower limits of force; when the force developed was too low or high, a beep signal was emitted to alert the participant that a force correction was needed.

## 4. Statistics

The normal distribution of the data was tested with the Shapiro–Wilk method. Descriptive statistics are expressed as the mean ± standard deviation. A repeated measures 2 × 2 ANOVA groups (control vs. training) × time (pre vs. post) was used to evaluate the effect of training. If any differences were significant, post hoc testing was performed (Tukey test) to identify which pair-wise comparisons between and within groups were significantly different from one another. The level of significance was set at *p* ≤ 0.05. The simple effect of training for each participant was defined as a relative increase in an analyzed variable observed after training, compared to the value observed before training, calculated with the following formula:

(1) $$\mathrm{RI}\left[\%\right]=\frac{x_{post}-x_{pre}}{x_{pre}}\;\times \;100$$

where RI is the relative increase, and *x* is the value of a given variable, measured before (*pre*) and after (*post*) training. We also calculated the lower and upper limits of the 95% confidence intervals (95% CI) for each relative increase. The effect size (ES) of the training was calculated with the paired two-sample *t*-test, and according to Goulet-Pelletier and Cousineau [22], we determined Cohen’s d with Hedges’ *g* correction. The scale presented by Cohen indicated that d < 0.41 represented a small ES, d = 0.41–0.70 represented a moderate ES, and d > 0.70 represented a large ES [23].

## 5. Results

The training was well tolerated by participants, and no one had an injury or a health problem following training (except for DOMS, occurring in all participants from the T group in the first week of exercise only). Moreover, attendance at all sessions was strictly monitored and was 98.6%.

There were no significant differences among the two groups in pretraining values of the variables. In the training group, 6 weeks of inertial training induced significant increases in muscle strength, compared to baseline, in both the impaired limbs (from 32 to 68%) and unimpaired limbs (from 31 to 64%). In contrast, muscle strength changes in controls were not significantly different from baseline. The percent increases in strength were also significantly greater in the training group than in the control group (Table 2). Moreover, high ES values were achieved in the training group, which indicated that inertial training was highly effective.

Breast-cancer-related lymphedema was evaluated with the L-Dex index. We found no significant changes from baseline (pre–post, *p* >0.05) in either group. Moreover, the percentage changes in L-Dex did not differ significantly in the training (6.32%) and control (1.16%) groups. However, it should be noted that the training group achieved a higher ES (0.29; 95% CI = −0.50−1.12) than the control group (0.04; 95% CI = −0.76–0.84).

The body composition did not change in either group during the 6 weeks training. The fat mass, muscle mass, and water content remained constant in both groups (Table 3).

The DASH questionnaire results indicated that the ability to perform specific daily activities, compared to baseline (pre-post), increased significantly in the training group, but not in controls. DASH scores decreased by 24.5% (ES = 0.52; 95% CI = −0.29–1.38) in the training group and by 3.99% (ES = 0.31; 95% CI = −0.49–1.14) in the control group. However, the percentage changes in DASH scores were not significantly different in the training and control groups. The training group showed significant improvements (*p* < 0.05) in the abilities to carry a shopping bag or briefcase (ES = 0.52; 95% CI = −0.28–1.35), carry a heavy object (over 10 lb) (ES = 0.42; 95% CI = −0.38–1.24), and wash the back (ES = 1.05; 95% CI = 0.21–1.94). Moreover, difficulty sleeping due to arm, shoulder, or hand pain significantly decreased in the training group (ES = 0.80; ES = 1.05; 95% CI = −0.02–1.65; *p* < 0.05). In the control group, the changes were not significant, except the ability to carry a shopping bag or briefcase, which decreased significantly (ES = −1.11; 95% CI = −2.01−0.27).

## 6. Discussion

The effectiveness of inertial training/rehabilitation in women after a mastectomy has not been studied to date. The present study showed that inertial training promoted significant improvements in the strength of all targeted muscle groups. The relative changes in muscle strength noted in the training group were greater than those observed in the control group, consistent with findings from previous studies that evaluated the efficacy of inertial training in older individuals. For example, Brzenczek-Owczarzak et al. [15] showed that, among older women, 4 weeks of inertial training improved the shoulder muscle strength from 3.5 to 21.9%. Moreover, among physically inactive older residents of a nursing home, 6 weeks of inertial exercises significantly increased (37.1–69.1%) the strength of elbow and knee flexor and extensor muscles [16]. Onambele et al. [24] found that 12 weeks of inertial training led to a significant increase in quadriceps strength and power in older individuals. It should be noted that, due to the specificity of our group, we used smaller loads than those used in the mentioned studies. We found that 6 weeks of inertial training improved the muscle strength (31–68%). This improvement was greater than that achieved with 20 weeks of mixed aerobic/resistance training (12%) among women that experienced a mastectomy [10]. Another study showed that 8 weeks of traditional resistance exercises performed by women that underwent a mastectomy induced significant increases in strength during the shoulder raise (22%), biceps curl (16%), triceps kickback (37%), and shoulder press (50%) [9]. Moreover, Hagstrom et al. [25] showed that 16 weeks of resistance training performed by women after a mastectomy increased the chest muscle strength by 20%. Therefore, our findings suggested that, among women that underwent a mastectomy, muscle strength improvements were similar or greater than those achieved with more traditional modalities. However, future studies are needed to confirm these findings.

Inertial training can increase the muscle strength by promoting muscle hypertrophy, improving the stretch-shortening cycle, increasing the excitability threshold of the Golgi tendon organs, and/or improving neuromuscular coordination [18]. We did not observe significant changes in muscle mass; this was probably due to the short duration of the program (6 weeks), and little influence of the small muscle groups trained over the body muscle mass. Therefore, the muscle strength was probably enhanced by changing the bioelectric muscle activity and improving neuromuscular coordination. This hypothesis was supported by results from other studies that found that electromiographic signals recorded from trained muscles increased significantly after inertial training [18,26,27]. However, future studies are needed to investigate this conjecture.

Inertial training improved some quality-of-life aspects related to physical function. The present study showed that the DASH scores decreased by 24.5% in the training group, which represented a significant improvement. Moreover, difficulties in performing some daily activities decreased significantly in the training group (e.g., the ability to carry a shopping bag or briefcase, carry a heavy object, and wash the back). It should be noted that sleep quality also improved in the training group. Similar results were reported by Ohira et al. [3], who showed that 6 months of traditional weight training significantly improved the quality of life of women after a mastectomy, based on the Physical Activity Readiness Questionnaire. Additionally, McKenzie and Kalda [7] concluded that 8 weeks of resistance training improved the quality of life among women, based on the SF−36 questionnaire.

Of note, four patients in the training group reported after the intervention that they experienced less or no pain in the shoulder joint when raising the arms above the head. Three patients reported that their flexibility had increased in the scar and that upper limb movements did not cause discomfort. However, we did not test the mechanical properties of the scar. Moreover, all subjects in the training group reported that inertial training was a pleasant form of exercise, and they particularly liked the ability to visualize the strength developed.

One negative consequence of a mastectomy is that lymphedema occurs in about 20% of treated women [28]. However, the prevalence depends on the treatment type. Some older reports suggested that strength training performed by women after a mastectomy could induce lymphedema [29,30]. However, the National Lymphedema Network stated that patients with lymphedema should perform all three main types of exercise—aerobic, strength, and flexibility—which should form part of a healthy lifestyle and is essential for effective lymphedema management [11]. Patients with or at risk of lymphedema should perform aerobic and resistance exercises [11]. The present study results showed that 6 weeks of inertial training did not lead to lymphedema in women that underwent a mastectomy (i.e., breast-cancer-related lymphedema did not change significantly after training). Therefore, inertial training appeared to be safe and beneficial for improving the limb strength without the risk of the arms becoming swollen. These findings were consistent with results from other studies that used other resistance training methods in women after a mastectomy [7,31,32,33,34]; they found no increase in lymphedema after resistance exercises. However, it should be noted that, although the changes in lymphedema after training were not significant, the ES was only 0.29, which is a small effect size. Moreover, we could not rule out the possibility that inertial training for more than 6 weeks might induce breast-cancer-related lymphedema. Future studies are needed to address this issue more comprehensively.

Inertial training did not influence the body composition in the tested women. None of the tested parameters (fat mass, upper limb muscle mass, water content) changed significantly. Previous studies showed that the muscle mass could increase with inertial training [18,26,27]. Naczk et al. reported that the muscle mass increased in young men by 10–15% following 5 weeks of inertial training performed three times per week. However, four muscle groups were trained (knee and elbow flexors and extensors), and participants developed their maximum strength during the training. In other studies, Tesch et al. [26] showed a 7.7% increase in quadriceps volume in middle-aged men, and Seyness et al. [27] observed a significantly increased quadriceps cross-sectional area after inertial training in young men. Herrero et al. [35] noted a 3% significant increase in muscle mass after 8 weeks of aerobic/resistance training in women post-mastectomy; however, resistance training included 11 exercises engaging the major muscle groups (chest press, shoulder press, leg extension, leg curl, leg press, leg calf rise, abdominal crunch, low back extension, arm curl, arm extension, and lateral pull-down); in our study, we trained only the arms and shoulders. Our findings were similar to those of Buchan et al. [36] and Dos Santos et al. [37], who found no effects of training on lean mass.

## 7. Limitations of the Study

The main study limitation was the small group of participants, which limited our ability to draw strong conclusions. Other limitations were the short duration of the intervention, selection of small muscle groups, and only focusing on the upper limbs. Moreover, there was the lack of data on longitudinal effects. Unfortunately, it was not possible to perform follow-up measures of 3 and 6 months after the project was completed. It would be interesting to investigate how long the training effect might be maintained.

## 8. Conclusions

Inertial training induced significant improvements in the strength of the shoulder flexors, extensors, abductors, and adductors in women that underwent a mastectomy. Inertial training also significantly improved the quality of life among trained women. Finally, we demonstrated that a 6 week inertial training did not cause breast-cancer-related lymphedema. These findings suggested that inertial exercises could be a useful rehabilitation method for women after a breast cancer treatment.

## Figures and Tables

**Table 1:** Treatment applied in training group (T) and control group (C).

Group	Simple Mastectomy	Breast-Conserving Surgery	Radiotherapy	Chemotherapy	Hormone Therapy	Lymphadenectomy
T	7	5	7	6	9	8
C	9	3	7	8	7	9

**Table 2:** Absolute values of strength (N), strength changes (%), and ES in tested shoulder muscles.

Group/Muscle		Flexion		Extension		Abduction		Adduction	
		N-IL	IL	N-IL	IL	N-IL	IL	N-IL	IL
T	Before	34.85 ± 9.55	34.81 ± 8.56	38.33 ± 10.51	37.15 ± 11.97	31.67 ± 8.25	30.98 ± 6.90	30.57 ± 7.67	29.73 ± 8.36
	After	43.50 ± 9.09	44.04 ± 10.69	53.04 ± 7.04	50.15 ± 10.61	41.82 ± 11.28	41.99 ± 12.37	47.81 ± 6.73	46.27 ± 8.28
	% changes	30.52 *, #	31.92 *, #	49.14 *, #	46.96 *, #	36.57 *, #	38.86 *, #	63.86 *, #	68.06 *, #
	ES	0.86	0.89	1.53	1.07	0.95	1.02	1.57	1.85
	95% CI	0.02–1.80	0.04–1.83	0.57–2.67	0.20–2.06	0.10–1.91	0.16−2.00	0.60–2.72	0.82–3.11
C	Before	30.07 ± 9.07	28.76 ± 6.65	32.63 ± 11.98	30.89 ± 8.69	26.60 ± 7.27	27.21 ± 6.19	27.72 ± 7.35	27.28 ± 6.81
	After	29.27 ± 7.09	29.09 ± 7.09	31.15 ± 10.33	32.01 ± 8.35	27.49 ± 7.42	26.66 ± 8.29	27.59 ± 11.55	29.01 ± 9.75
	% changes	0.20	2.59	−1.46	3.18	4.34	−2.25	−0.07	8.34
	ES	−0.09	0.04	−0.12	0.12	0.11	−0.09	−0.01	−0.01
	95% CI	−0.90–0.70	−0.85–0.75	−0.93–0.67	−0.67–0.93	−0.68–0.92	−0.86–0.71	−0.81–0.79	−1.00–0.60

Notes: *—significant difference from baseline, #—significant difference from the control, (*p* ≤ 0.05). N-IL—non-impaired limb, IL—impaired limb.

**Table 3:** Body composition changes following 6 weeks of intervention.

Group/Muscle		Fat Mass (%)	Fat Mass (kg)	Total Body Water (kg)	Upper Limb Muscle Mass (kg)
	Before	37.62 ± 4.78	28.59 ± 7.81	32.58 ± 4.00	4.36 ± 0.59
	After	37.98 ± 4.50	28.60 ± 7.79	32.13 ± 3.99	4.32 ± 0.59
T	% changes	1.42	1.68	−0.33	−1.03
	ES	0.07	0.00	−0.1	−0.06
	95% CI	−0.72–0.88	−0.79–0.80	−0.91–0.70	−0.87–0.73
	Before	36.25 ± 2.48	25.72 ± 3.90	31.68 ± 3.07	4.58 ± 0.60
	After	36.88 ± 2.77	26.31 ± 3.59	31.71 ± 3.92	4.52 ± 0.59
C	% changes	0.74	−0.15	−1.25	−1.60
	ES	0.22	0.14	0.01	−0.09
	95% CI	−0.57–1.04	−0.65–0.96	−0.79–0.81	−0.90–0.70

## Data Availability

The data presented in this study are available on request from the corresponding author.
